# Quantitative proteomics for the analysis of *Plasmodium falciparum* and its red blood cell host - a preliminary study

**DOI:** 10.1186/1475-2875-13-S1-P74

**Published:** 2014-09-22

**Authors:** Patrícia Machado, Tiago R Vaz, Fátima Nogueira, João Rodrigues, Licínio Manco, Letícia Ribeiro, Ed Bergstrom, David Ashford, Rui Vitorino, Jane Thomas-Oates, Jerry Thomas, Ana P Arez

**Affiliations:** 1 Instituto de Higiene e Medicina Tropical, Universidade Nova de Lisboa, Lisboa, Portugal; 2 Centro de Investigação em Antropologia e Saúde, Universidade de Coimbra, Coimbra, Portugal; 3 Departmento de Hematologia, Centro Hospitalar de Coimbra, Coimbra, Portugal; 4 Centre of Excellence in Mass Spectrometry, University of York, York, UK; 5 Centro de Espectrometria de Massa, Universidade de Aveiro, Aveiro, Portugal

## Background

Malaria is a major cause of death and has been one of the strongest selective forces on the human genome selecting variants that influence pathogenesis, host response and may protect against disease severity [1, 2, 3]. Previous studies suggest the association between malaria and red blood cell (RBC) glucose-6-phosphate dehydrogenase (G6PD) and pyruvate kinase (PK) deficiencies in humans [4]. This study focuses on RBC-pathogen interactions and the effect of these two enzymatic deficiencies on parasite development. Proteomic information from Plasmodium infection is scarce, and proteomes of both G6PD- and PK-deficient RBC and from parasites growing in these cells have not yet been characterized. We performed a proteomic study to detect the relative abundance of proteins from both G6PD- and PK-deficient RBC, and also from Plasmodium infecting these cells. It will provide key information about malaria dynamics, but also about enzyme deficiencies causing important haemolytic anaemia. Furthermore, it will contribute to a better understanding of host-parasite interactions.

## Materials and methods

*P. falciparum* 3D7 was maintained in continuous synchronous cultures. Invasion ratios and maturation ratios were determined. Samples were prepared for proteomic analysis by FASP and GelC-MS. LC-MS and CID-MS/MS of peptides were carried out by nano-RP-LC-MS/MS. Data were analyzed for identification of peptides and quantification through comparison of normalized peak area/intensity of each identified peptide.

## Results

Only results from parasite proteome are available so far. There was an over-expression of defensive molecules against oxidative stress (heat shock proteins and chaperones) in parasites growing in G6PD-deficient RBC, and an under-expression of global proteins (mostly proteins involved in haemoglobin catabolism and trafficking/RBC remodelling) in parasites growing in PK-deficient RBC. The study will still look into infected RBC proteome and assess the influence of these alterations in the putative protective effect against malaria.
